# Attention-deficit hyperactivity disorder in spontaneously hypertensive rat strain SHR/NCrl is associated with specific expression of uncoupling proteins, glucose transporter 1 and BACE1

**DOI:** 10.3389/fncel.2025.1612751

**Published:** 2025-08-18

**Authors:** Tsunehisa Sato, Rolf Schreckenberg, Klaus-Dieter Schlüter

**Affiliations:** ^1^Physiologisches Institut, Justus-Liebig-Universität, Giessen, Germany; ^2^Department of Anesthesiology and Intensive Care, Hamamatsu University of Medicine, Hamamatsu, Japan

**Keywords:** physical activity, medulla oblongata, ADHD animal models, HIF, neuroinflammation

## Abstract

Attention-deficit hyperactivity disorder (ADHD) is the most prevalent neurodevelopmental disorder worldwide. To improve treatment strategies against ADHD a better understanding of underlying pathophysiology is required. Spontaneously hypertensive rats (SHR) from the strain SHR/NCrl are a suitable rodent model of ADHD. Here we compared the gene expression in the brains of SHR/NCrl strain to that of other genetically related hypertensive and normotensive rat strains that do not show an ADHD phenotype. In addition, the impact of physical activity on genes that display such differences was also addressed because high physical activity is one non-pharmacological option to cure ADHD symptoms. RNA was isolated from the medulla oblongata, the olfactory bulb, and the cortex. Gene expression was analyzed by qRT-PCR. The cortical expression of GLUT1 was also analyzed by Western Blot. Physical activity was improved by free access to running wheels for six months. Female rats were used in this study and sacrificed at the age of 7.5 months. The results show that gene expression in SHR/NCrl differs from other SHR strains in the olfactory bulb, medulla oblongata, and the cortex. Main differences were obtained for *SLC25A14*, coding for the protein UCP5, *SLC2A1*, coding for the protein glucose transporter (GLUT) 1 in the cortex and *CCL2* and for *BACE1* in the medulla oblongata. The expressions of *SLC25A14* and *BACE1* in the medulla oblongata were normalized in physical active rats. Our study further underlines the usefulness of the SHR/NCrl strain as an ADHD animal model when combined with proper controls. Furthermore, this study identifies genes that are specifically down-regulated in the medulla oblongata of SHR/NCrl and that are affected by activity status.

## 1 Introduction

Attention-deficit hyperactivity disorder (ADHD) is one of the most common chronic psychiatric disorders in children and adolescents that often persist into adulthood (Xu et al., 2018). However, the knowledge about the neurobiological basis of this disorder is uncomplete and this limits any improvement of successful treatment strategies. The spontaneously hypertensive rat, strain SHR/NCrl, is the best validated animal model for ADHD as it shows symptoms of inattention, hyperactivity, and impulsivity (Custudo et al., 2023). Analysis of the transcriptome of these rats may help to identify pathophysiological mechanisms contributing to ADHD. However, such type of analysis requires proper control strains. As such the gene expression may be compared to genetically related SHR strains, as SHR/KyoRj and SHR/NHsd because these strains do not develop symptoms of ADHD. To discriminate between the effect of hypertension and ADHD the use of a proper normotensive control strain is also mandatory. As such controls, normotensive Wistar rats are superior compared to Wistar Kyoto rats (WKY/NCrl) because WKY/NCrl rats chare some but not all behaviors related to ADHD (Dela Pena et al., 2015). NKY/NCrl Wistar rats display inattention whereas Wistar rats do not have such a phenotype (Dela Pena et al., 2015). Using a rodent ADHD model reduced neurotransmission was identified as one potential mechanism contributing to ADHD (Custudo et al., 2023). Furthermore, comparison of the expression profile between SHR/NCrl, WKY/NCrl and Wistar rats revealed additional genes differentially regulated in the prefrontal cortex. The cortex is involved in attention and therefore this information may give a hint to attention deficit disorder. The genes that were identified were linked to transcription, synaptic transmission, and immune responses (Dela Pena et al., 2015; Dela Pena et al., 2017a). These genes are also responsive to amphetamine administration (Dela Pena et al., 2017a; Dela Pena et al., 2017b).

Using SHR/NCrl metabolic differences were also identified between this strain and non-ADHD strains. Furthermore, oxidative metabolism was affected (Dupuy et al., 2021). It is likely to assume that such metabolic changes are caused by different gene regulation. However, the identification of such molecules is still lacking. We hypothesized that uncoupling proteins or genes related to metabolism in a broader sense are contribute to the differences between the ADHD model SHR/NCrl and other strains without such symptoms. Uncoupling proteins (UCPs) show a unique expression profile in the brain that differs from UCP expression in other tissues. Among the five UCP isoforms that are expressed in rats, four are constitutively expressed in the brain. The brain expresses a large amount of UCP2, a smaller amount of UCP3, but also two untypical isoforms of UCP that are specifically expressed in the brain, namely *SLC25A27*, coding for UCP4, and *SLC25A14*, coding for UCP5 (Lengacher et al., 2004; Mao et al., 1999; Smorodchenko et al., 2009). The latter ones are not found in other tissues of these animals. The precise function of UCPs is not well understood and may show tissue-specific and strain-specific variations (Kutsche et al., 2022). Nevertheless, all these proteins are located in the inner mitochondrial membrane and affect substrate exchange between the cytoplasma and the mitochondrial matrix as well as oxidative stress generated by mitochondria. We addressed the expression of UCPs in our study as these proteins may affect metabolism and oxidative stress, two potential candidates that participate in ADHD development (Dupuy et al., 2021; Dimatelis et al., 2015; Kozlowska et al., 2019). In addition to uncoupling proteins, receptor-dependent pathways that involve the local renin-angiotensin-system (RAS) or endothelin system may add oxidative stress to neurons (Veerasingham and Raizada, 2003; Kuwaki et al., 1994). Hyperactivity of the local RAS can reduce cognition (Jackson et al., 2018). In addition to the classical symptoms of ADHD such as inattention, hyperactivity, and impulsivity also proteins linked to Alzheimer Disease may play a role in ADHD symptomatic (Zhang et al., 2015). Therefore, we also analyzed the expression of genes related to memory as they are described in the context of Alzheimer’s disease. ADHD is associated with an increased risk of dementia (Levine et al., 2023).

The various parts of the brain exert different functions. In the light of ADHD we focused in this study on the cortex that is involved in generating attention, on the olfactory bulb, the most important distance sense in rodents, and the medulla oblongata. That was done because defects in sensing may also contribute to attention and cognition deficits and the olfactory bulb represents the most important distance sense of rats (Franca et al., 2020; Franca et al., 2022). Finally, we analyzed the expression in the medulla oblongata as these rats are hypertensive and the medulla oblongata is involved in central control of blood pressure.

Exercise has recently been identified in these models as a non-pharmacological procedure to cure some of the ADHD symptoms (Franca et al., 2020). Running performance to mimic physical exercise attenuated spatial orientation and social interaction impairments (Robinson et al., 2012) and alleviated hyperactivity in rats (Kim et al., 2011). Interestingly, exercise can also affect the expression of uncoupling proteins that are dysregulated in different neurological disease including Parkinson disease (Tsai et al., 2020). Therefore, it was our interest to investigate the effect of physical activity on differentially regulated genes in SHR/NCrl. In conclusion, this study compared the expression profiles of genes related to key events of metabolism and oxidative stress in a suitable model of ADHD.

## 2 Materials and methods

The investigations are in agreement with the “Guide for the Care and Use of Laboratory Animals” purchased by the U. S. National Institute of Health (NIH Publication No. 85–23, revised 1996). The study was approved by the local authorities (RP Gießen; V 54–19 c 20 15 h 01 GI 20/1 Nr. 76 and GI 20/1 Nr. 77/2014).

### 2.1 Animal model

The current project aimed at studying the expression profile of genes in the brain of different commercial available SHR strains (SHR/NHsd, SHR/KyoRj, SHR/NCrl), and normotensive Wistar rats (RjHan: Wi). Moreover the impact of high physical activity as performed by voluntary running wheel activity was addressed. Our interest came up as one out of these four strains has a unique phenotype, namely ADHD. Here we used exclusively female rats for the following reasons: First, female rats display a higher voluntary running wheel activity compared to male rats and therefore allow a better analysis of the effect of running. Second, female rats display an age-dependent degree of mitochondrial number but the quality of mitochondria is improved by up-regulation of UCP4 and UCP5, to proteins in the focus in this study (Guevara et al., 2009). Third, restriction to one sex reduces variability in experimental data and therefore allows us to use fewer animals. In conclusion, scientific and ethical reasons motivated us to restrict our analysis to female rats.

After 7.5 months all rats were anesthetized by isoflurane inhalation. After cervical dislocation, brains were prepared and the cortex, medulla oblongata, and olfactory bulb were extracted and immediately transferred to fluid nitrogen and stored at -80°C until use.

### 2.2 Analysis of physiological parameters

Rats had free access to running wheels at the age of six weeks thus prior to the onset of hypertension in SHRs. Running wheels were connected to a computer and the duration of using wheels was recorded as well as the total distance. From these data we calculated the run performance (expressed as km per week) for each rat of the running groups and the average speed (km/h). In summary, experiments started in the pre-hypertensive state of six weeks and lasted for another six months.

Two weeks prior to the end of the experiments some rats from each group were randomly selected and the blood pressure and heart rate were analyzed using a tail-cuff method as described before (Braun et al., 2018).

### 2.3 Rt-PCR

Total RNA was isolated from brains using peqGOLD TriFast according to the manufacturer’s protocol. Genomic DNA was removed by treatment of samples with 1 U DNase/μg RNA for 15 min at 37°C. One microgram of RNA was used in a 10 μL reaction to synthesize cDNA unsing Superscript RNase H Reverse Transcriptase and oligo(dt) as primers. Sequences of primers used a summarized in Supplementary Table 1. In general we analyzed the expression of uncoupling proteins (*UCP1, UCP2*, *UCP3*, *SLC25A27*, *SLC25A14*), renin-angiotensin system (*AGTR1*, *AGTR2*, *ACE1*, *ACE2*, *REN*), endothelin system (*EDNRB*, *ECE1*, *EDN1*), metabolism (*SLC2A1*, *SLC2A4*), inflammation (*CCL2*, *IL6*), oxidative stress (*SOD2*, *CAT*), Alzheimer Disease-associated proteins (*PSEN1*, *PSEN2*, *RAG*, *SG2*, *BACE1*), and hypoxic stress related proteins (*HIF2A*, *VEGFA*). Quantification was based on the ΔΔC_T_ method and performed as described before (Livak and Schmittgen, 2001). Neuron-specific enolase (*NSE*) was used for normalization as it showed no differences in the expression in the cortex, medulla oblongata, and olfactory bulb (*p* = 0.590; two-sided one-way ANOVA with Student–Newman–Keuls *post hoc* analysis).

### 2.4 Western Blots

Total protein was extracted from isolated cortex by lysis buffer (Cell Signaling, Technology, Frankfurt, Germany), according to the manufacture’s protocol. Protein concentration was adjusted to 40 μg/μl. The expression of GLUT1 was analyzed with an antibody directed against provided by Samuel W. Cushman (NIH, National Institute of Diabetes and Digestive and Kidney Diseases, Bethesda, Montgomery, MD, USA). Expression was normalized to the expression of beta-Actin identified with a pan-specific actin antibody (A2668; Sigma, Saint Louis, Missouri, USA). Secondary antibodies (horseradish peroxidase-coupled secondary antibody) directed against rabbit IgG were perchased from Dako (Agilent Technologies, Santa Clara, USA).

### 2.5 Statistics

Data are expressed as means ± S. D. with original data points given in the figure or presented as size effects with 5 and 95% confidence intervals. *p* values were calculated by ANOVA with Student–Newman–Keuls *post-hoc* analysis. Effect Sizes were analyzed by Cohen’s d. SPSS 27 was used to calculate these data.

## 3 Results

### 3.1 Animal characteristics

**Figure 1:**
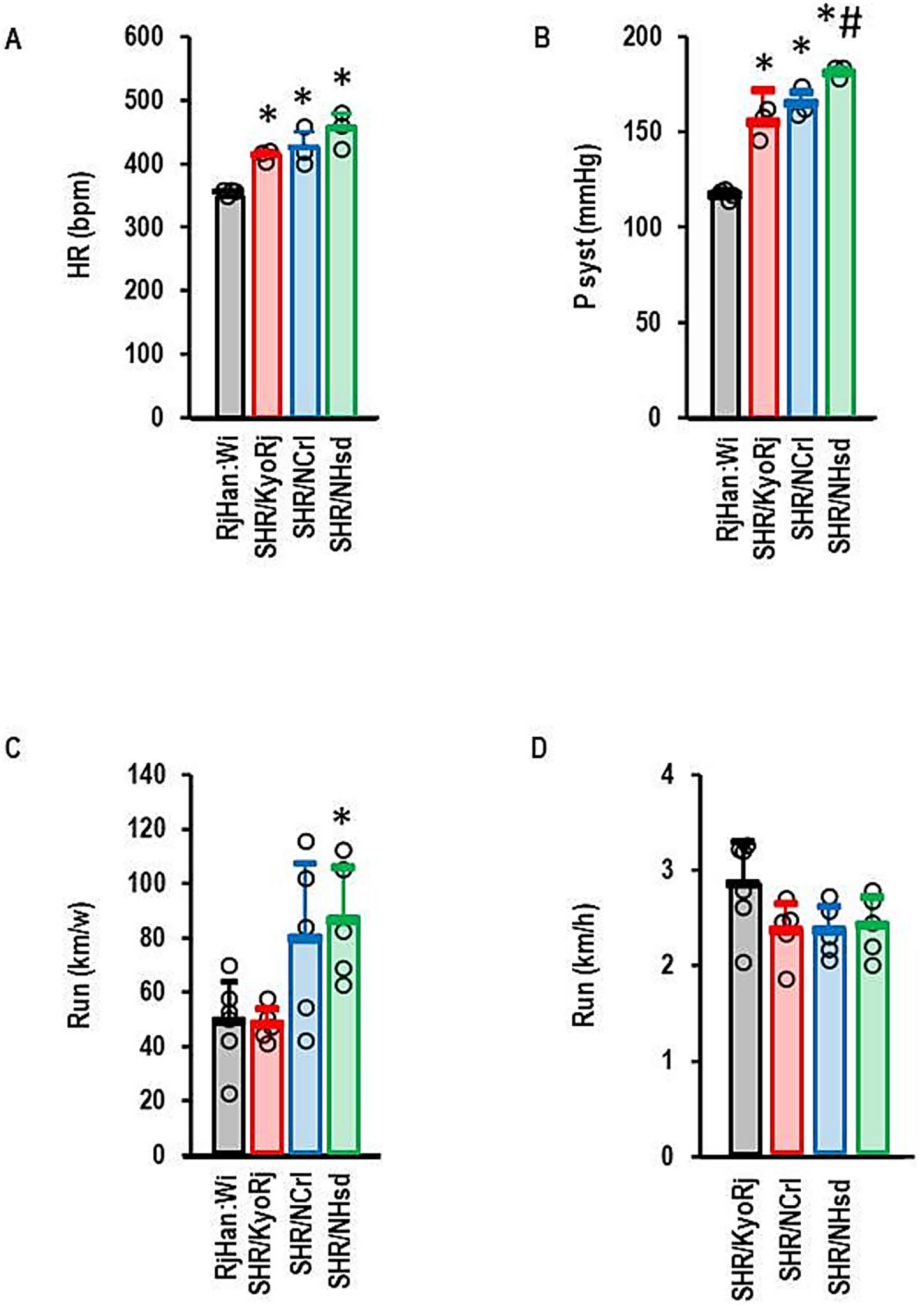
Physiological parameters of rat strains used in this study. **(A)** Resting heart rate (HR) in beats per minute (bpm); **(B)** Systolic blood pressures (P syst) in mmHg; **(C)** Total running activity per week; **(D)** Velocity of running in wheels. Data are means ± S. D. with individual data points. One-Way ANOVA with Student–Newman-Keul’s *post hoc* analysis. *, *p* < 0.05 vs. RjHan: Wi **(A–C)**. A + B: RjHan: Wi (*n* = 4), SHR strains all *n* = 3; C + D: *n* = 5 for all SHR strains and *n* = 6 for RjHan: Wi.

In this study we used a normotensive Wistar strain and three different commercially available SHR strains (SHR/NCrl, SHR/KyoRj, and SHR/NHsd). Among them, SHR/NCrl represents an established ADHD model. All SHR strains had higher resting heart rates than normotensive rats with no strain-dependent differences among SHR strains (Figure 1A). All three SHR strains were hypertensive compared to the normotensive Wistar rat with SHR/NHsd showing the highest values (Figure 1B). Voluntary running activity differed between the three SHR strains. SHR/KyoRj showed the lowest running motivation (Figure 1C). In contrast to the total amount of running activity, running velocity was not different between the three strains (Figure 1D). The ADHD model strain SHR/NCrl was more comparable to SHR/KyoRj when comparing the blood pressure but more comparable to SHR/NHsd considering running motivation.

### 3.2 Expression of UCPs

**Figure 2:**
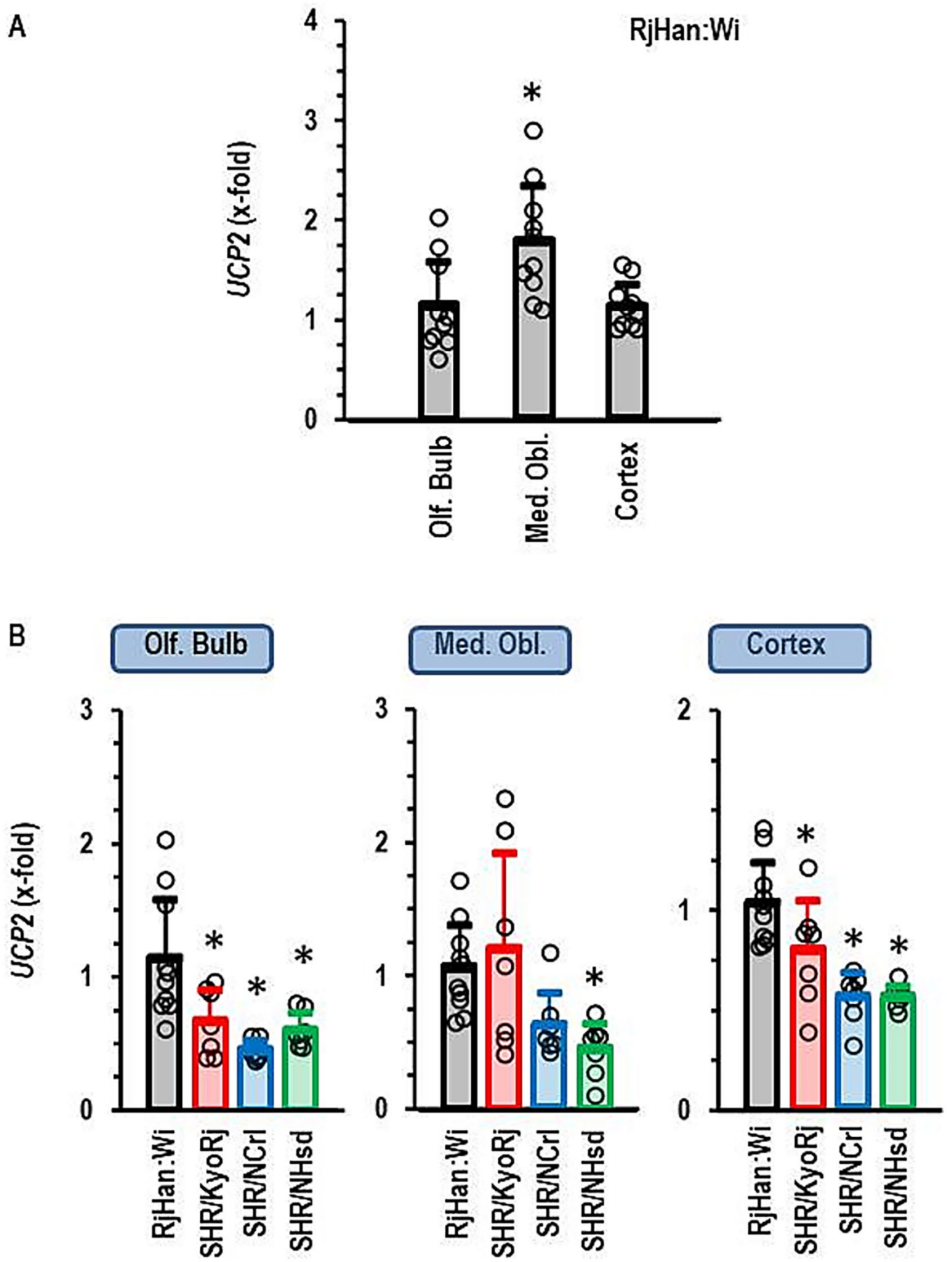
Expression of *UCP2* in the brain. **(A)** Differential expression in three different brain regions in normotensive Wistar rats (RjHan: Wi, *n* = 10 each). The expression level was normalized to the median of the expression in the olfactory bulb (Olf. Bulb). **(B)** Differential expression in the brain regions between strains. Data are means ± S. D. with original data points. One-Way ANOVA with Student–Newman-Keul’s *post hoc* analysis. *p < 0.05 vs. Olf. Bulb **(A)** or RjHan: Wi **(B)**. *n* = 7 for all SHR strains.

First, we proved constitutive expression of *UCP2* in all parts of the brain that were investigated here. This comparison of local expression was done with brains from normotensive Wistar rats. *UCP*2 was indeed expressed in all parts with higher expression in the medulla oblongata than in the cortex or olfactory bulb (Figure 2A). Next, we analyzed the expression of *UCP2* in SHR strains and compared it to that in Wistar rats. In the olfactory bulb and the cortex, *UCP2* was down-regulated in all SHR strains (Figure 2B). In the medulla oblongata it was not repressed in SHR/KyoRj (Figure 2B).

**Figure 3:**
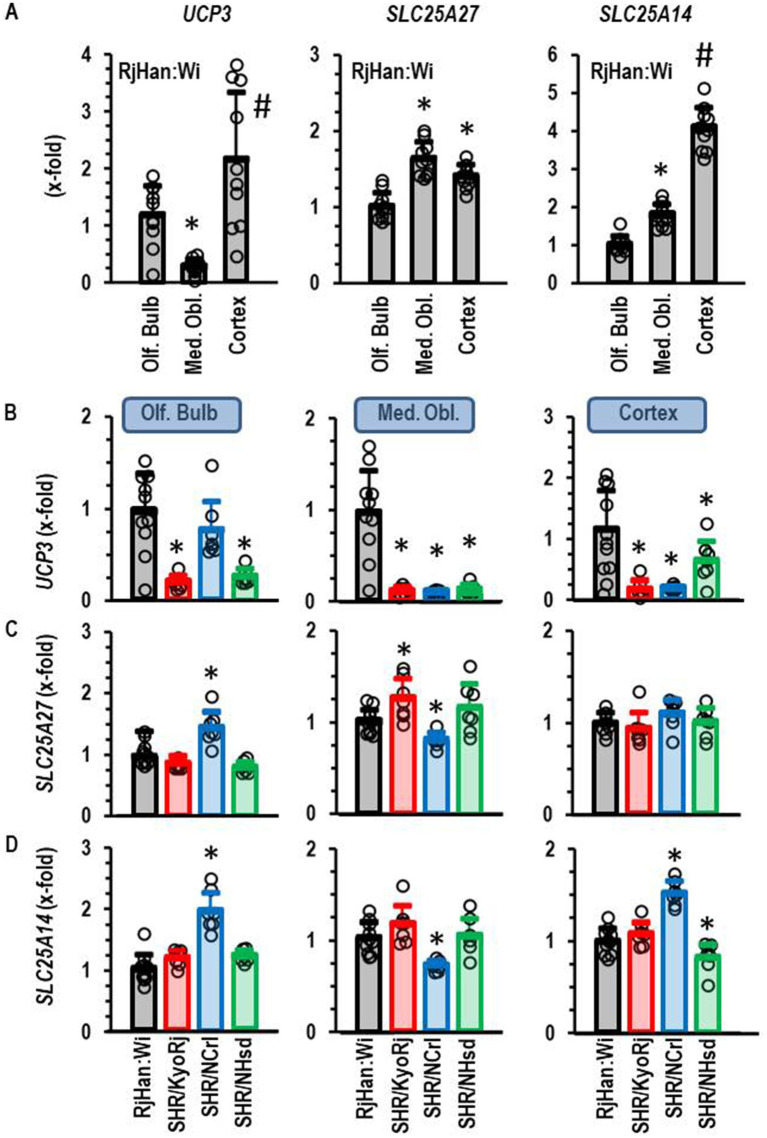
Expression of *UCP3*, *SLC25A27*, and *SLC25A14* in the brain. **(A)** Differential expression in three different brain regions in normotensive Wistar rats (RjHan: Wi; *n* = 10). The expression level was normalized to the median of the expression in the olfactory bulb (Olf. Bulb). **(B–D)** Differential expression in the brain regions between strains. Data are means ± S. D. with original data points. One-Way ANOVA with Student–Newman-Keul’s *post hoc* analysis. *, *p* < 0.05 vs. Olf. Bulb **(A)** or RjHan: Wi **(B–D)**. *n* numbers as in Figure 2.

Subsequently, we performed a similar type of analysis for the four other isoforms of uncoupling proteins, namely *UCP1*, *UCP3*, *SLC25A27*, and *SLC25A14*. *UCP1* was not expressed in any of the three parts of the brain and it was not further investigated. *UCP3*, *SLC25A27*, and *SLC25A14* were constitutively expressed in all parts of the brain. The expression of each of these three isoforms was higher in the cortex than in the olfactory bulb (Figure 3A). The expression of *SLC25A27* and *SLC25A14* was also higher in the medulla oblongata than in the olfactory bulb, whereas *UCP3* was less expressed in the medulla oblongata than in the olfactory bulb or cortex (Figure 3A). Next we investigated the expression of these UCP isoforms in SHR strains (Figures 3B–D). The ADHD reference strain SHR/NCrl showed a unique expression profile for these uncoupling protein isoforms. All three isoforms were higher expressed in the olfactory brain of this strain than in the other two SHR strains. *SLC25A27* and *SLC25A14* were also stronger expressed as in the olfactory brain of normotensive Wistar rats. *UCP3*, *SLC25A27* and *SLC25A14* were down-regulated in SHR/NCrl in comparison to the other rat strains in the medulla oblongata. Finally, *SLC25A14* was stronger expressed in the cortex and olfactory bulb of SHR/NCrl than in any other rat strain. In conclusion, the ADHD model strain SHR/NCrl showed a unique expression profile for untypical UCP isoforms that are only expressed in the brain.

### 3.3 Expression profile of other genes

**Figure 4:**
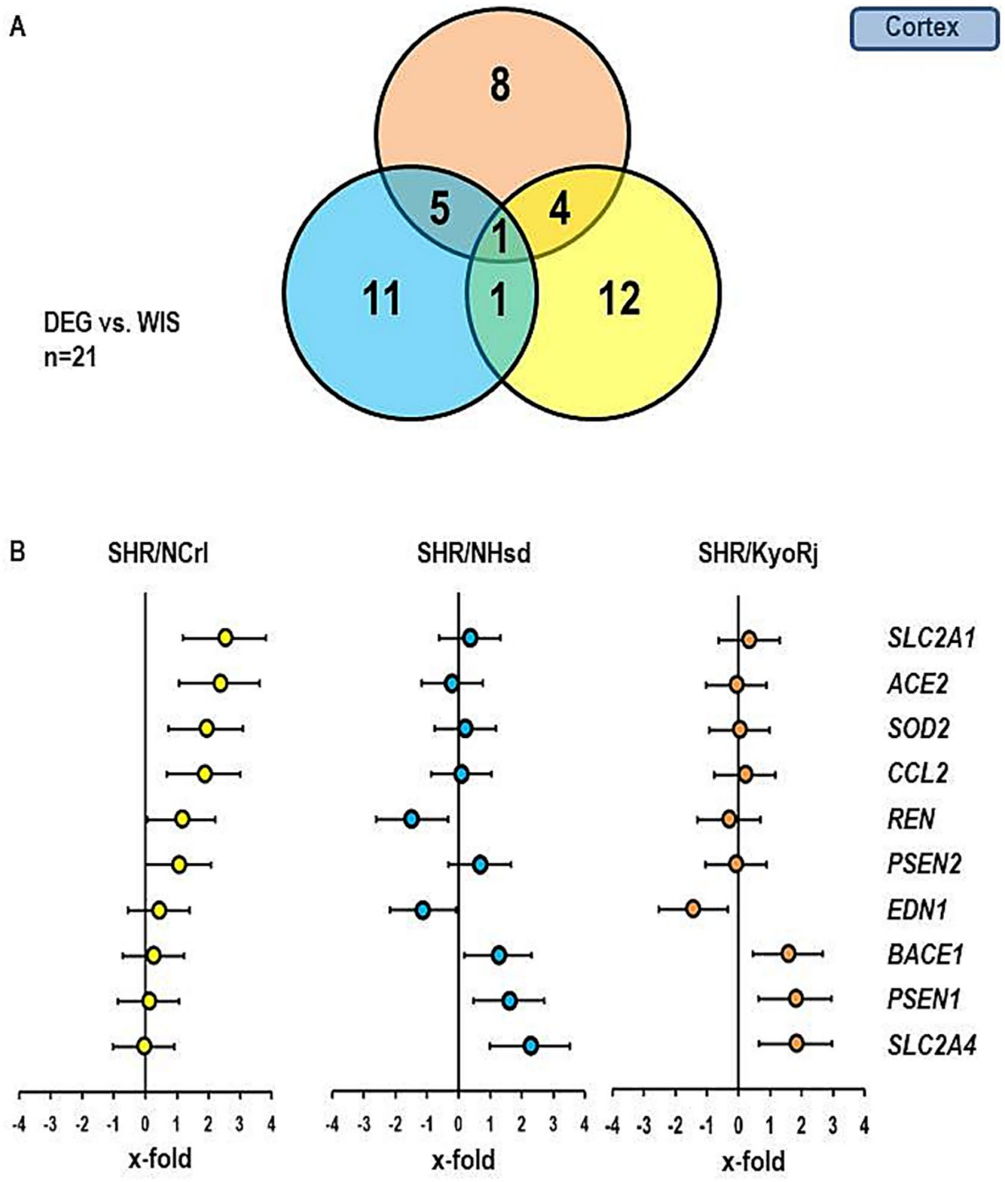
Comparison of the gene expression profile in the cortex between the three SHR strains and RjHan: Wi. **(A)** Venn-diagram showing the number of differentially regulated genes in comparison to RjHan: Wi. **(B)** Expression of ten genes differentially expressed in SHR strains vs. RjHan: Wi in the three strains. Data represent the effect sizes and the 5 and 95% confidence interval. Data for SHR/NCrl are shown in yellow, data for SHR/NHsd are shown in blue, and data for SHR/KyoRj are shown in brown.

In the next step we extended our analysis to genes that are linked to the local renin-angiotensin system, inflammation, local endothelin system, and memory function. The Venn-Diagram in Figure 4 gives an overview about the different regulation of these genes in the cortex. The data show that in comparison to normotensive rats the highest number of differentially expressed genes (DEG) was found in the SHR/NCrl strain (*n* = 12). More important, there was little overlap between SHR/NCrl and the other two strains (*n* = 4 versus SHR/KysRj and *n* = 1 versus SHR/NHsd). These data indicate a unique expression profile in the cortex of SHR/NCrl. In Figure 4B the expression profile of 10 genes is given that were differentially expressed between the three SHR strains. We found a strong induction of *SLC2A1*, *ACE2*, *SOD2*, and *CCL2* versus normotensive Wistar rats in the ADHD reference strain SHR/NCrl. Importantly, these differences did not occur in the two other SHR strains. In contrast we found an induction for *BACE1*, *PSEN1*, and *SLC2A4* in the cortex of SHR strains not linked to ADHD that were absent in SHR/NCrl.

**Figure 5:**
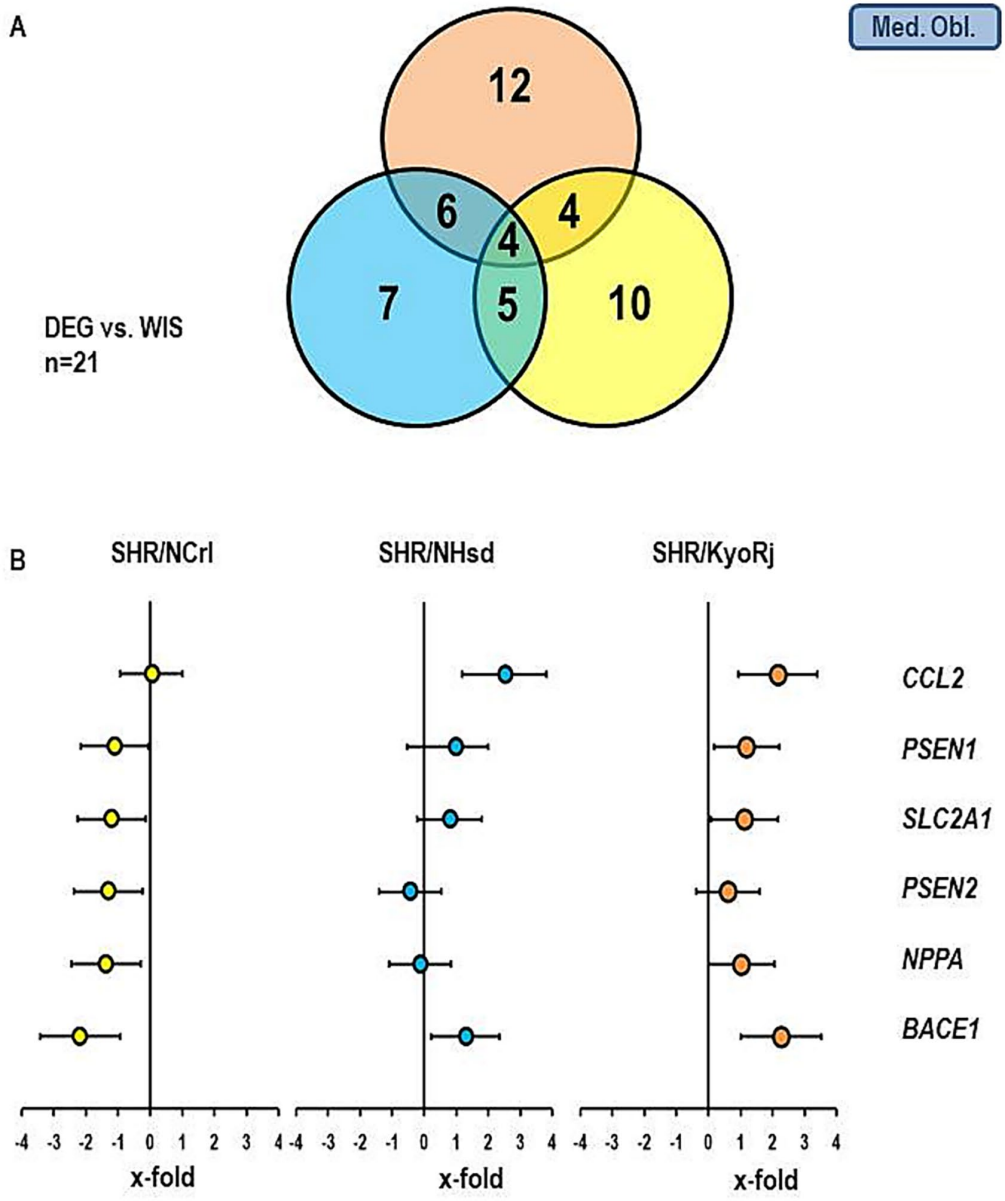
Comparison of the gene expression profile in the medulla oblongata between the three SHR strains and RjHan: Wi. **(A)** Venn-diagram showing the number of differentially regulated genes. **(B)** Expression of six genes differentially expressed in SHR strains. Data represent the effect sizes and the 5 and 95% confidence interval. Data for SHR/NCrl are shown in yellow, data for SHR/NHsd are shown in blue, and data for SHR/KyoRj are shown in brown.

**Figure 6:**
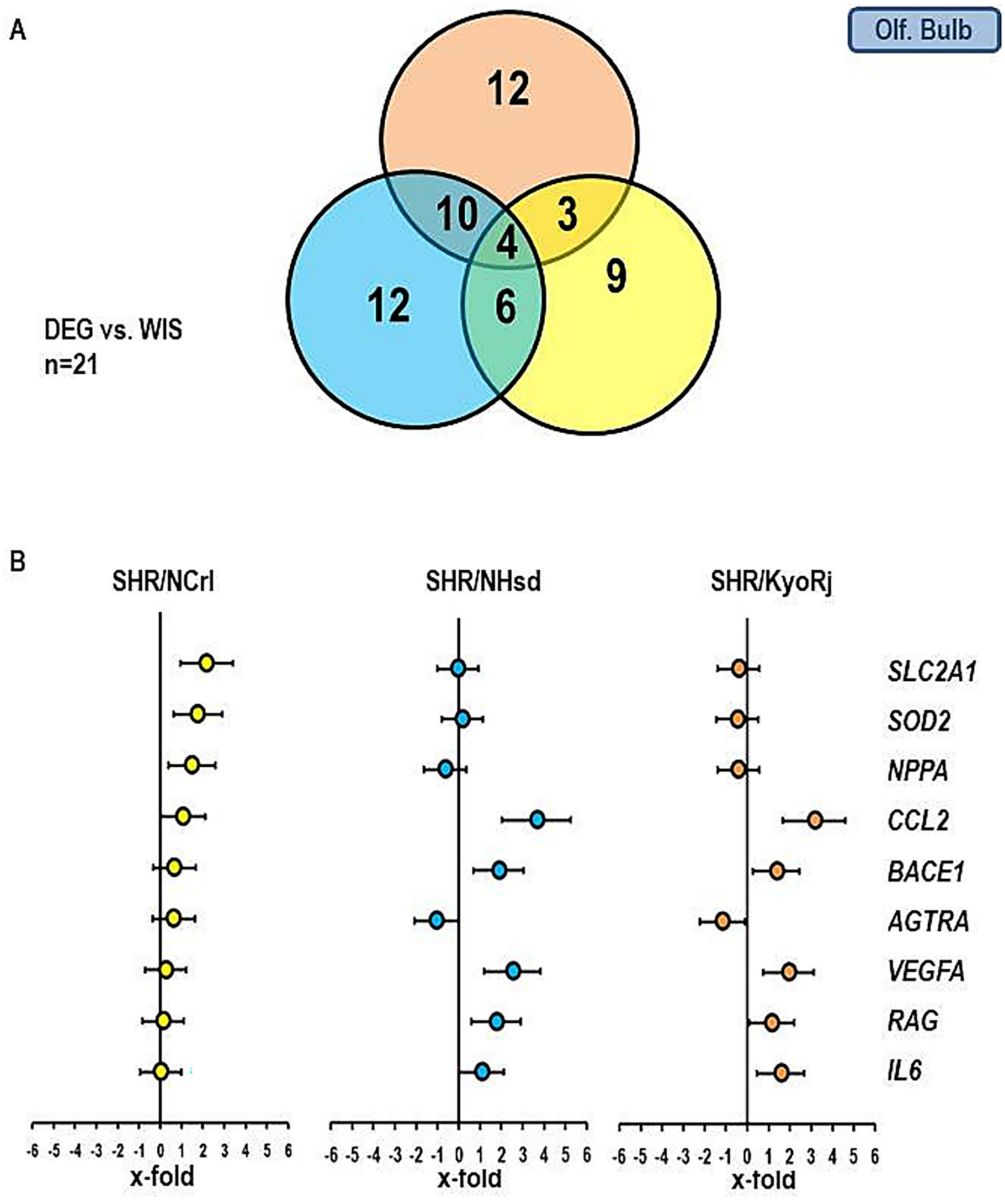
Comparison of the gene expression profile in the olfactory bulb between the three SHR strains and RjHan: Wi. **(A)** Venn-diagram showing the number of differentially regulated genes. **(B)** Expression of nine genes differentially expressed in SHR strains. Data represent the effect sizes and the 5 and 95% confidence interval. Data for SHR/NCrl are shown in yellow, data for SHR/NHsd are shown in blue, and data for SHR/KyoRj are shown in brown.

Figures 5, 6 show similar analysis for the medulla oblongata and olfactory bulb. In the medulla oblongata the main difference between SHR/NCrl and the other two SHR strains is that the up-regulation of *BACE1* as seen in SHR/KyoRj is replaced by a strong down-regulation in SHR/NCrl. In the olfactory bulb an up-regulation of *SLC2A1* is absent in the two non-ADHD SHR strains.

**Figure 7:**
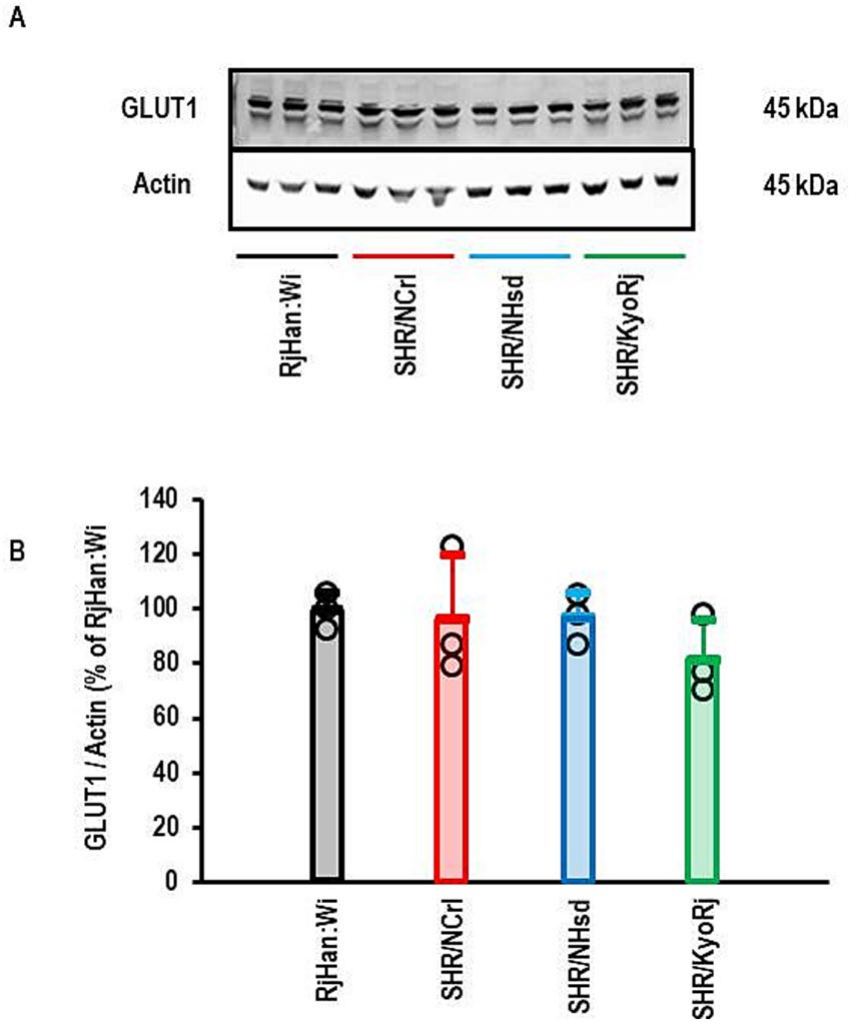
Western Blot analysis of the expression of GLUT1 in the cortex. **(A)** Representative Western Blot showing the expression of GLUT1 and actin that was used to normalized the data for loading variance. **(B)** Quantification of the Blot shown in **(A)**. Data are means ± S. D. with original data points. One-Way ANOVA with Student–Newman-Keul’s *post hoc* analysis (*p* > 0.05).

The data presented above show that the up-regulation of *SLC2A1* in the cortex of SHR/NCrl is the strongest difference in comparison to normotensive rats and specific for this SHR strain. Subsequently, we analyzed the corresponding protein expression of GLUT1. As indicated in Figure 7, the strong increase in *SLC2A1* expression in SHR/NCrl is not translated into more protein as there are no differences between the groups.

### 3.4 Effect of voluntary running wheel activity

**Figure 8:**
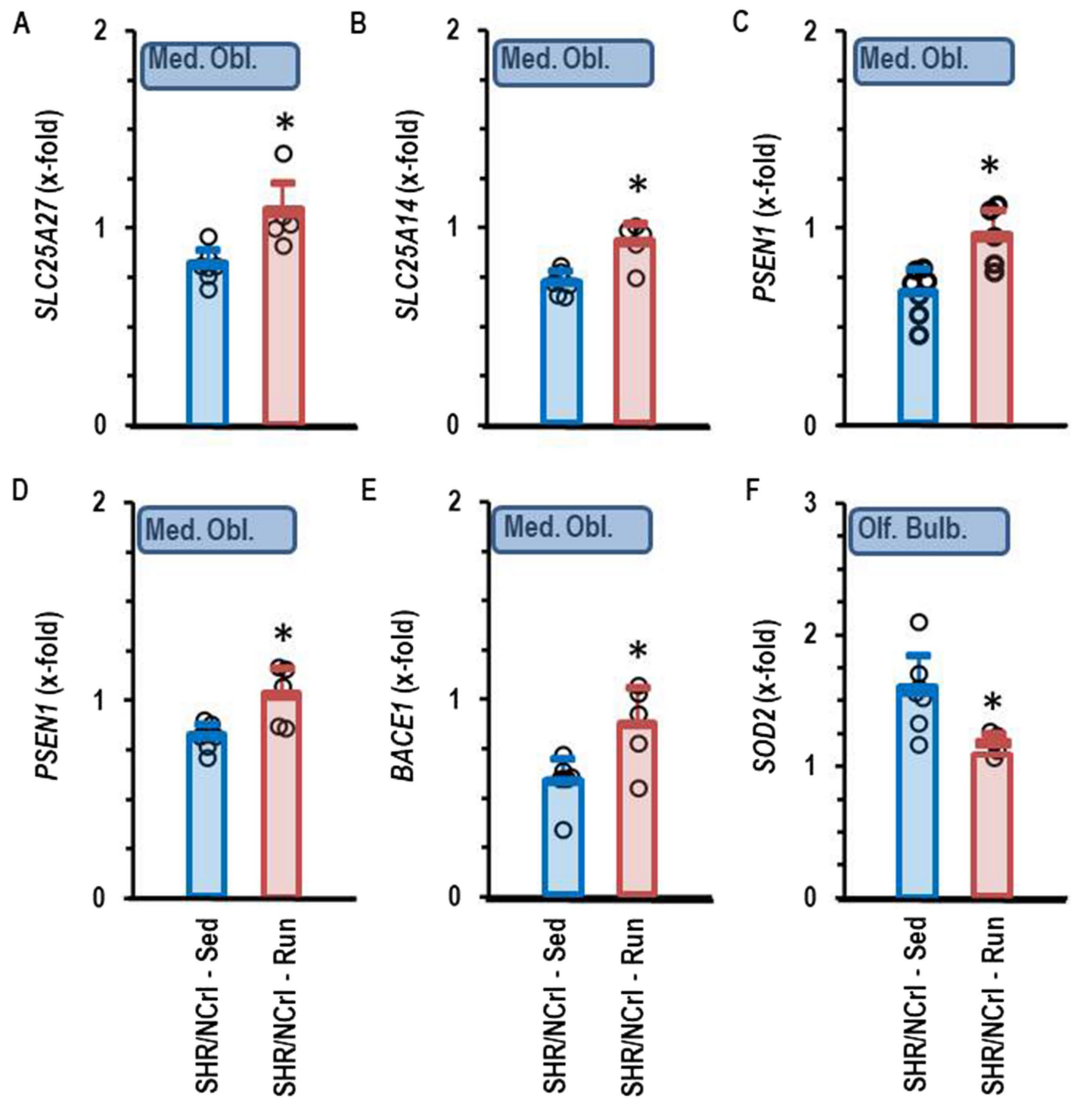
Comparison of gene expression profile in sedentary SHR/NCrl (Sed) and SHR/NCrl with voluntary running wheel activity (Run). Data are means ± S.D. with original data points (*n* = 7 for sedentary and *n* = 5 for run. Data are normalized to the expression of RjHan:Wi. *, *p* < 0.05 vs. sedentary. Data show the following genes of the Medulla oblongata: SLC25A27 **(A)**, SLC25A14 **(B)**, PSEN1 **(C)**, PSEN2 **(D)**, and BACE1 **(E)** and for the olfactory bulb: SOD2 **(F)**.

Voluntary running wheel activity of SHR/NCrl rats normalized the expression of five genes in the medulla oblongata that were normally down-regulated in this strain. These are two brain-specific uncoupling proteins (*SCL25A27* and *SLC25A14*) and three genes linked to Alzheimer Disease (*PSEN1*, *PSEN2*, and *BACE1*; Figures 8A–E). Furthermore, voluntary running wheel activity normalized the expression of *SOD2* in the olfactory bulb (Figure 8F). However, running wheel activity did not modify the mRNA expression of genes in the cortex.

## 4 Discussion

SHR/NCrl, a suitable rodent model for ADHD research, differs from other SHR strains and from normotensive rats with respect to gene expression. As discussed in detail otherwise such a conclusion depends mainly on the selection of suitable reference strains (Sagvolden et al., 2009). Therefore, we compared in this new study SHR/NCrl strains with three different strains, two hypertensive rat strains and one normotensive rat strain. Both SHR strains have a hypertensive phenotype in common with SHR/NCrl and showed increased resting heart rate. However, both strains do not show symptoms related to ADHD. Therefore, the strain SHR/NCrl differs from these rats and from the normotensive rats. Importantly, we used a Wistar rat strain different from Wistar Kyoto rats because Wistar Kyoto rats show inattention which is part of the ADHD phenotype and may therefore not count as an optimal reference strain.

Our data confirmed that SHR/NCrl displays a unique gene expression in comparison to non-ADHD rat strains but there are several new aspects in our study: First, this new study focused on uncoupling proteins, second, it exerts the effect of physical activity on specific gene expression in SHR/NCrl, and third it compares the expression profile in three different parts of the brain. The main new finding of our study is that among the UCPs, *SLC25A14*, coding for the protein UCP5, is up-regulated in the cortex and olfactory bulb in SHR/NCrl but down-regulated in the medulla oblongata. This expression profile is unique among the three different SHR strains. Down-regulation in the medulla oblongata but not the up-regulation in the olfactory bulb and cortex were normalized in physical active SHR/NCrl. Furthermore, the expression profile of *SLC2A1* in SHR/NCrl differed from all other rat strains analyzed here. Nevertheless, alterations in *SLC2A1* expression are not directly linked to ADHD as the protein expression remained unchanged. Such a finding suggests either a different rate of protein turnover or a lack of sufficient translation. One may speculate that protein turnover is energy consuming. This may at least indirectly affect neuronal function a suggestion that requires future attention.

In our study we analyzed differential expression of genes between hypertensive and normotensive rats. Some of the genes under investigation were blood pressure-dependent regulated, i.e., *REN* in the medulla oblongata and *UCP2* and *UCP3* that were down-regulated in all parts of the brain and in all three SHR strains versus normotensive rats. However, other genes showed a unique expression profile in SHR/NCrl and normotensive Wistar rats as well as the two other SHR strains. These genes a not linked to hypertension. It is attractive to hypothesize that these genes are potential candidates that contribute to the ADHD phenotype of SHR/NCrl. Our data show that such differences can be observed in different parts of the brain. They are mainly linked to metabolism.

As outlined above, the expression of *SLC25A14*, coding for UCP5, showed a unique expression profile in SHR/NCrl. UCPs in the brain are important for neuroprotection, metabolism, and oxidative stress (Andrews et al., 2005; Cardoso et al., 2015). They are dysregulated in several neurodegenerative diseases (Cardoso et al., 2013; Guzman et al., 2010; Montesanto et al., 2018; Ramsden et al., 2012; Thangavel et al., 2017; Tsai et al., 2020; Wu et al., 2010). However, this study is the first study that associates a differential expression of *SLC25A14* with ADHD. A high expression of UCP4 and UCP5 is associated with better differentiated mitochondria that can compensate an age-dependent reduction in mitochondria number in female rats (Guevara et al., 2009). Mechanistically, UCP5 expression in neurons is regulated by PGC1α and this is linked to reduced oxidative stress (Han et al., 2020). However, PGC1α co-regulates the expression of *SOD2*, *UCP2*, and *UCP4* in neurons whereas our study shows a selective up-regulation of *SLC25A14*. This does not support the above mentioned hypothesis that PGC1α triggers the high expression of *SLC25A14* in SHR/NCrl rats. Furthermore, the co-regulation of several genes involved in oxidative defense does not allow us to conclude that the main function of UCP5 is oxidative defense. Interestingly, detailed ex vivo analysis has shown that the three main isoforms of UCP expressed in the brain, namely UCP2, UCP4, and UCP5 transport H^+^ and Cl^−^ across the mitochondrial membrane. In addition they differ in the way how fatty acids activate transport function of these isoforms (Hoang et al., 2012; Hoang et al., 2015). Thus, the up-regulation of *SLC25A14*, as shown in this study, may suggest indeed a different metabolism in the brains of SHR/NCrl. A trigger for UCP5 expression in the brain is hypoxia (Viggiano et al., 2016). We found indeed a strict co-regulation with hypoxia-dependent regulated *SLC2A1* in the cortex and olfactory bulb. Two further hypoxia-regulated genes were induced, namely *HIF2* and *VEGFA*. In summary the most important information we concluded from this part of the study is: In SHR/NCrl rats the expression of atypical but brain-specific UCP isoforms is altered in comparison to both normotensive rats and SHR strains without ADHD phenotype. The expression differs stronger in the olfactory bulb from other strains than in other parts of the brain. A down-regulation of *SLC25A14* in the medulla oblongata may contribute to metabolic stress in this part of the brain. High physical activity normalized the expression of *SLC25A14* in the medulla oblongata.

In the cortex we found an induction of *CCL2* expression in SHR/NCrl. This may indicate an inflammatory phenotype this part of the SHR/NCrl rat. However, *CCL2* was also increased in other parts of the brain in SHR strains without ADHA phenotype when compared to normotensive rats. In addition, we observed a down-regulation of *NPPA* in SHR/NCrl rats in the medulla oblongata. Generally this is linked to an increased risk of pressure load in brain vessels (Carnio et al., 2004).

The strongest effect of physical activity was seen in the medulla oblongata where the expression of five differentially regulated genes of SHR/NCrl was normalized. Here, we observed an effect of physical activity on the expression of genes associated to Alzheimer Disease, such as *BACE1*. The link between altered expressions of genes associated with dementia in ADHD model systems may lead to possible explanations why the risk for dementia is higher in patients with adult ADHD (Levine et al., 2023). A recent paper has already highlighted the relationship between the expression of *BACE1* and early ADHD syndrome in drosophila indicting an evolutionary old relationship (Zhang et al., 2015). However, we found a strong induction of *BACE1* in both SHR strains without ADHD and low expression of *BACE1* in the olfactory bulb that could be normalized by physical activity. Thus, we observed in the rats an inversed gene regulation than expected from former studies. In this context it is important to remind that *BACE1* has multiple functions in the body and brain. More important, genetic deletion of *BACE1* in mice showed a hyperactive phenotype (Kandalepas and Vassar, 2014). This corresponds to our finding of low *BACE1* expression in SHR/NCrl.

In conclusion, our study reveals a possible role for UCP5, GLUT-1, and BACE1 in the onset of ADHD in the SHR/NCrl strain. The data are based on the transcriptional regulation of these genes in SHR/NCrl vs. other SHR strains and normotensive rats in different parts of the brain. Finally, the data suggest that at least in the medulla oblongata regulation is sensitive to physical activity. By comparison of SHR/NCrl with three different rat strains we minimized the risk of misinterpretation of data by selection of reference strains. Furthermore, the focus on uncoupling proteins and the observation which effects may be reversible by high physical activity are important new steps to improve our current understanding about the physiological basis of ADHD.

## Data Availability

The original contributions presented in the study are included in the article/Supplementary material, further inquiries can be directed to the corresponding author/s.
