# Supplementary material for: Attention-deficit hyperactivity disorder in spontaneously hypertensive rat strain SHR/NCrl is associated with specific expression of uncoupling proteins, glucose transporter 1 and BACE1
Source: Front Cell Neurosci. 2025 Aug 18;19:1612751. doi: 10.3389/fncel.2025.1612751 (PMC12399616; doi:10.3389/fncel.2025.1612751)
Supplement: Supplementary file 1 [file Table_1.DOCX]

Supplement Table 1: List of Primers used in the study

Name forward reverse NCBI

ACE1 ACAGCTATAACTCGAGTGCCG ACAGCTCCTTGGCCTTCTTG NM_012544.1

ACE2 ACAATTGTTGGAACGCTGCC CGCTTCATCTCCCACCACTT NM_001012006.2

AGTR1 GAAGTCTCGCCTTCGCCGCA CAGCCCTATGGGGAGCGTCG NM_030985.4

BACE1 CCACAGACGCTCAACATCCT CCATGAGGGATGCTCACCAG NM_019204.2

CAT TTTTCACCGACGAGATGGCA CTGACTCTCCAGCGACTGTG NM_012520.2

CCL2 TCACGCTTCTGGGCCTGTTGT TCCAGCCGACTCATTGGGATCA NM_031530.1

ECE1 TCTGGCCAACATCACCATCC TAGACCACGATGGGCTCAGA NM_053596.2

EDN1 CCGTATGGACTAGGAAGCCC TGCATGGTACTTTGGGCTCG NM_012548.2

EDNRB GCTAGCCATCACTGCGATCT TGTCTTGGCCACTTCTCGTC NM_017333.1

HIF2A GGGTACGTGAGGCATGTTGA CCGTCGGTCAGACCAGAAAA NM_024359.1

IL6 CACTTCACAAGTCGGAGGCT TCTGACAGTGCATCATCGCT NM_012589.2

NPPA ATGGGCTCCTTCTCCATCAC TCTTCGGTACCGGAAGCTG NM_012612

NSE GGAACAAGTTGGCCATGCAG TCTCCAGGATATTGGGGGCA AF019973.1

PSEN1 ATGCTGGTCGAAACAGCTCA GGTACCCTCCTTTGGGCTTC NM_019163.4

PSEN2 ACTCCGTGCTGAACACTCTG AGGAGCATCAGGGAGGACAT NM_031087.2

rAG TAAGCGATGCCCTTCTCGTG CTGCATGTCTCTTTGGCAACA X07648.1

REN GAGGCAGTGACCCTCAACAT GTGTCCACCACTGCCATACA NM_022177.3

SG2 ATACACCAGGCTCAACAGGC ATCTCCTTGCACACTGTCGG X13231.1

SLC2A1 GCTGTGGCTGGCTTCTCTAA CCGGAAGCGATCTCATCGAA NM_138827.1

SLC2A4 ACCGTCTTCACGTTGGTCTC ATCAAGATGGCACAGCCACA NM_012751.2

SLC25A14 GCAGCAGTGATTGTAAGCGG ACAGGGAAAGTGCCGAACTC NM_053501.3

SLC25A27 GCCGATGTCATCAAAAGCCG CCCTGCAAATCGCAGTCTCAT XM_006247484.3

SOD2 ATGTTGTGTCGGGCGGCGTG TCGCGTGGTGCTTGCTGTGG NM_017051

UCP2 CACCGTCATTGCCTCCCCCG CGGAGCATGGTCAGGGCACA NM_019354.2

UCP3 GACCCACGGCCTTCTACAAA TCAAAACGGAGATTCCCGCA NM_009464.3

VEGFA TGCCCCTAATGCGGTGTGCG GGCTCACAGTGAACGCTCCAGG NM_001171624.2
